# Presence of toxic microbial metabolites in table olives

**DOI:** 10.3389/fmicb.2015.00873

**Published:** 2015-08-28

**Authors:** Eduardo Medina-Pradas, Francisco Noé Arroyo-López

**Affiliations:** Food Biotechnology Department, Instituto de la Grasa, Consejo Superior de Investigaciones CientíficasSeville, Spain

**Keywords:** microbial risk, foodborne pathogens, table olives, mycotoxins, *Clostridium*, biogenic amines

## Abstract

Table olives have an enormous importance in the diet and culture of many Mediterranean countries. Albeit there are different ways to produce this fermented vegetable, brining/salting, fermentation, and acidification are common practices for all of them. Preservation methods such as pasteurization or sterilization are frequently used to guarantee the stability and safety of fermented olives. However, final products are not always subjected to a heat treatment. Thus, microbiota is not always removed and appropriate levels of acidity and salt must be obtained before commercialization. Despite the physicochemical conditions not being favorable for the growth of foodborne pathogens, some illness outbreaks have been reported in the literature. Street markets, inappropriate manipulation and storage conditions were the origin of many of the samples in which foodborne pathogens or their metabolites were detected. Many authors have also studied the survival of pathogens in different styles of table olive elaboration, finding in general that olive environment is not appropriate for their presence. Inhibitory compounds such as polyphenols, low availability of nutrients, high salt content, low pH levels, bacteriocins, or the addition of preservatives act as hurdles against undesirable microorganisms, which contribute to obtaining a safe and good quality product.

## Production of Table Olives

The fermentation of olive fruit has many centuries of history, particularly in the Mediterranean basin, where this fermented vegetable has had a great influence on the culture and diet of many countries. According to the last consolidated statistics of the International Olive Council, worldwide production currently exceeds 2.4 million tons per year. Spain, Turkey, Egypt, Syria, Algeria, Greece, and Morocco are among the main producers, albeit Argentina, Peru, and USA are also important contributors (International Olive Council [IOC], 2015). Thus, table olive processing is spread worldwide and represents an important economic source for olive-growing countries.

Olive fruit cannot be consumed directly from the tree due to its peculiar characteristics (presence of the bitter glucoside compound oleuropein, high fat, and low sugar content). For this reason, diverse methods were developed to make them palatable. Although many of them share the general process of brining/salting, fermentation, and acidification, they can differ slightly between areas of production. The Trade Standard Applying to Table Olives (International Olive Council [IOC], 2004) defines table olives as: ‘*the product obtained from suitable olive cultivars, processed to remove their natural bitterness, and preserved (by natural fermentation, heat treatment or preservatives) with or without brine until consumption*.’ Among the most important table olive industrial processing methods we can find: (i) the so-called Spanish-style (alkali treated green olives), which represent about 50–60% of production, (ii) the so-called Californian-style (ripening of olives by alkaline oxidation), and (iii) directly brined olives (green, changing color or naturally black fruits) (Garrido-Fernández et al., 1997).

In all table olive processing methods described above, microorganisms have an important role, determining the safety, quality and flavor of the final product. Lactic acid bacteria (LAB) and yeasts are considered beneficial microorganisms, opposite to the role played by *Enterobacteriaceae* and *Propionibacteriaceae* (Garrido-Fernández et al., 1997; Arroyo-López et al., 2012; Hurtado et al., 2012). Traditionally, olive fermentation occurred spontaneously, but the process is not fully predictable and sometimes can lead to product spoilage or sanitary risks (Lanza, 2013). The present mini-review deals with the biological hazards posed by microorganisms in table olives, as well as the diverse hurdles that olive fermentation environments offer against growth of undesirable microorganisms.

## Sanitary Risks Caused by Microorganisms or Their Metabolites in Table Olives

Despite fermented table olives having a long history of microbial safety, diverse biological hazards may be present in the finished product (**Table 1**). Among the most relevant, we can mention:

**Table 1:** Summary of the main types of biohazards reported in table olives.

Type of biological hazard	Microorganism or compound detected	Type of table olives	Reference
Biogenic amines	Putrescine	“zapatera” green olives	Hornero-Mendez and Garrido-Fernández (1994)
		Greek-style olives	García-García et al. (2001)
	Cadaverine		Tofalo et al. (2012)
	Tyramine		
Mycotoxins	Ochratoxin	“Greek-style” black olives	Gourama and Bullerman (1988)
			El Adlouni et al. (2006)
	Citrinin		Ghitakou et al. (2006)
			Franzetti et al. (2011)
	Aflatoxin B		
Presence of foodborne pathogenic bacteria	*Listeria monocytogenes*	Green olives Sliced black olives	Caggia et al. (2004) RASFF Portal (2012a)
	*Staphylococcus* sp.	Black olives	Asehraou et al. (1992)
		Brined olives	Pereira et al. (2008)
	*Coliforms*	Black olives	Asehraou et al. (1992)
		Green olives	Franzetti et al. (2011)
	*Yersinia* and *Escherichia coli*	Spanish-style olives	Lucena-Padrós et al. (2014)
	*Clostridium*	Black olives	Fenicia et al. (1992)
			Debord et al. (1920)
		Olives stuffed with almonds	Jalava et al. (2011)
		Green olives from Italy	Cawthorne et al. (2005) RASFF Portal (2012b)

### (i) Biogenic Amines

The consumption of foods containing high amounts of toxic biogenic amines may cause food intoxication and intolerance, with diverse associated symptoms such as migraines, headaches, depression, diarrhea, insomnia, etc., indicating the need for a better hygiene process. These compounds can be formed in table olives by spoilage microorganisms with amino acid decarboxylase activity. Hornero-Mendez and Garrido-Fernández (1994) reported the presence of biogenic amines (putrescine, cadaverine, and tyramine) in fermented green table olives with “zapatera” spoilage. The concentration of biogenic amines can increase during olive storage but the levels found in the final products are usually low and should not represent a health concern (García-García et al., 2001). Recently, Tofalo et al. (2012) also detected in naturally fermented olives a low quantity of biogenic amines, as well as the presence by RT-qPCR of biogenic amines producing bacteria.

### (ii) Mycotoxins

These compounds are secondary toxic metabolites produced by some species of mold (mainly *Aspergillus*, *Penicillium*, and *Fusarium* genera) under aerobic and humidity conditions (El Adlouni et al., 2006). Mycotoxins in foods can be of concern for consumers, causing disease in human and other vertebrates with symptoms such as skin irritation, immunosuppression, neurotoxicity, etc. Contamination of table olives with various types of mycotoxins (Ochratoxin, Aflatoxin B, and Citrinin) have been documented in cracked olives (Franzetti et al., 2011), but Greek-style black olives are the most affected (Gourama and Bullerman, 1988; Ghitakou et al., 2006). Fortunately, mycotoxin levels usually found in table olives are too low to cause disease.

### (iii) Foodborne Pathogenic Bacteria

Diverse works have reported the presence of *Listeria monocytogenes* (Caggia et al., 2004; RASFF Portal, 2012a), *Staphylococcus aureus* (Asehraou et al., 1992; Pereira et al., 2008), and *Enterobacteriaceae* species such as *Yersinia enterocolitica* and *Escherichia coli* (Asehraou et al., 1992; Franzetti et al., 2011; Lucena-Padrós et al., 2014) in table olives. However, there are no reports of illness outbreaks caused by these microorganisms in table olives. Botulism, associated with *Clostridium botulinum* growth, is certainly the most relevant biohazard in table olives. Diverse outbreaks associated with homemade table olives and recalls of suspected products have been reported (Debord et al., 1920; Fenicia et al., 1992; Cawthorne et al., 2005; Jalava et al., 2011; Pingeon et al., 2011; RASFF Portal, 2012b). It should be emphasized that artisanal productions or inadequate storage (pH ≥ 4.5 units) were often the origin of these outbreaks. **Table 2** shows the epidemiological cases of botulism reported in table olives.

**Table 2:** Major illness outbreaks associated with botulism in table olives.

Type of table olive	Reason	Relevance	Region/Country	Reference
Black olives	Improper sterilization	12 deaths	OH, USA	Debord et al. (1920)
		5 deaths	Detroit, MI, USA	
		4 deaths	NY, USA	
		7 deaths	TN, USA	
		1 death	CA, USA	
	Incorrect storage after opening	5 cases with 0 death	Italy	Fenicia et al. (1992)
Green olives	Incorrect homemade preparation (pH 6,2)	16 cases with 0 death	Molise, Italy	Cawthorne et al. (2005)
			Campania, Italy	
			Puglia, Italy	
Olives stuffed with almonds	Incorrect manufacturing by producer	2 cases with 1 death	Helsinki, Finland	Jalava et al. (2011)
Green olives paste	Incorrect thermal treatment by homemade producer	9 cases with 0 death	South–east and northern of France	Pingeon et al. (2011)

### (iv) Degradation of Organic Acids

Spoilage microorganisms associated with fermented vegetables such as *Lactobacillus buchneri* are able to produce acetic acid from lactic acid consumption under anaerobic conditions (Johanningsmeier and McFeeters, 2013), whilst *Propionibacterium* and *Pectinatus* species are able to convert lactic acid to propionic acid (Breidt et al., 2013; Lucena-Padrós et al., 2014). Oxidative yeasts can also consume the lactic and acetic acids produced during olive fermentation under aerobic conditions (Ruiz Cruz and González Cancho, 1969). However, they are not able to use these acids in the absence of oxygen. Lactic acid consumption in table olives reduces the preservative power of fermented olives and increases the pH values, which can allow for the growth of others undesirable microorganisms with the consequent loss of product quality and food safety.

## Table Olives: Hurdles against Biological Hazards

The olive fermentation process led by LAB involves the consumption of sugars to produce a wide range of final products with preservative effects; among the most important is lactic acid. These preservative compounds, together with low pH, protein and vitamin content, as well as reduced water activity (chloride salt is added to brine in a range of 5–11%), provide an acidic and salty environment which is adverse for the growth of undesirable microorganisms.

Other compounds excreted by microorganisms can also act as biopreservative agents. Bacteriocins are bacterial proteins or peptides that show a bactericide effect against closely related species. Jimenez-Díaz et al. (1993) isolated a bacteriocin producer *Lactobacillus plantarum* strain from green olive fermentation. The inhibitory compounds produced by this microorganism (plantaricins S and T) were active against bacteria that can cause spoilage in olive fermentations (*Propionibacteriaceae* and *Clostridium*) as well as natural competitors of *Lactobacillus plantarum* in olive fermentation brines (Ruíz-Barba et al., 1994). Likewise, yeasts produce toxic proteins or glycoproteins, also known as killer factors, are able to inhibit the growth of fungi and other non-desirable yeast species acting as biocontrol agents. *Debaryomyces*, *Pichia*, and *Candida* are genera with a considerable number of killer strains isolated from table olives (Hernández et al., 2008).

Table olive fermentations also contain antimicrobial compounds that limit the growth of LAB and others microorganisms, mainly in non-alkali treated olives (Medina et al., 2010). It has been recently demonstrated that some phenolic and oleosidic substances such as the dialdehydic form of decarboxymethyl elenolic acid (EDA), as well as EDA linked to hydroxytyrosol (Hy-EDA) present in olive brines, possess significant bacteriocide activity against foodborne pathogens, even greater than other phenolic compounds isolated from foods or synthetic biocides (Medina et al., 2009; Brenes et al., 2011). Thus, survival studies carried out with *E. coli* O157:H7 in Spanish-style table olive fermentation, show inhibition of the pathogen in all assayed conditions (Spyropoulou et al., 2001). Similar behavior was observed in the survival of *Bacillus cereus* in green olive fermentation, where the population declined steadily during the fermentation process (Panagou et al., 2008). Recently, Grounta et al. (2013) investigated the survival of diverse foodborne pathogens artificially inoculated on natural black table olives. They demonstrated that natural black olives are not a favorable environment to support the growth of the assayed pathogens, and the population of all them showed a rapid decline throughout the first 2 days of storage. Medina et al. (2013) studied the survival of diverse food-borne pathogens (*E. coli*, *Salmonella enterica*, *Listeria monocytogenes*, and *S. aureus*) in industrial olive brines from different cultivars (Manzanilla, Gordal, Hojiblanca, etc.). They found a correlation with the presence of polyphenols, considered inhibitory compounds from olives fruit. 5-log reduction of population inoculated was achieved between 5 min to 17 days in the least deleterious brine. Hence, according to the available data, table olive industrial brines of different olive cultivars and elaboration processes, do not constitute a favorable environment for any of the pathogenic bacteria tested.

## How to Reduce Biological Hazards in Table Olives

The objective of table olive producers should be to achieve zero risk in the case of illness and injury caused by toxic microbial metabolites. This can only be achieved by following practices that ensure that the fruits selected for processing are: produced under Good Agricultural Practices (GAP); processed under the principles of Good Manufacturing Practices (GMP) and produced at premises with equipment and personnel strictly following Good Hygienic Practices (GHP). All these requisites must be considered in the framework of food safety management systems, which include not only the HACCP System, but also other food defense tools to prevent intentional adulterations, i.e., CARVER (Criticality Accesibility Recuperability Vulnerability Effect Recognizability), TACCP (Threat Assessment and Critical Control Points), VACCP (Vulnerability Analysis and Critical Control Points).

In many cases, the fermentation of olive fruit still occurs spontaneously, which can sometimes lead to spoilage of the final product or to sanitary risks. In order to prevent these problems, the processing can be controlled through physicochemical (addition of acids, salt, temperature control, preservatives, or application of modified atmospheres) or microbiological approaches. To improve fermentation and consistently produce high quality, safe, final products; many authors have recommended strict process control of the above parameters, in addition to the use of starter cultures (see Corsetti et al., 2012 for a complete review on this aspect). The search for starters with application in olive fermentation and vegetables in general, has for many years, been focused on the activity of LAB and their technological applications. However, in the last decade, several publications have emphasized the importance of the role that selected yeasts can play when used as starter cultures during table olive processing (Arroyo-López et al., 2012; Bevilacqua et al., 2012, 2013). Moreover, the selection of microorganisms as starters in olive fermentation and vegetables in general, has been exclusively based on diverse technological criteria (homo-fermentative metabolism; high acidification rate and fast consumption of fermentable substrates; organic acids, polyphenols, high pH and salt tolerance; flavor development or production of bacteriocins) (Duran-Quintana et al., 1999; Sánchez et al., 2001; Corsetti et al., 2012; Hurtado et al., 2012; Di Cagno et al., 2013; Heperkan, 2013). However, in addition to technological characteristics, recent studies on the development of starter cultures for table olives have focused on the study of the probiotic potential of native microorganisms. These studies must include both LAB and yeasts for the development of a mixed-multifunctional starter, in order to improve and expand the form of action of the culture by the use of two complementary microorganisms with different properties.

## Conclusion

The harsh environmental conditions found in the fermentation process (low pH, high salt content, presence of inhibitory compounds, sugar consumption, etc.), and the presence of other additional hurdles (production of bacteriocins, killer factors, addition of preservatives, etc.), make table olives an adverse habitat for the development of foodborne pathogens. If such growth ultimately occurs, the presence of undesirable microorganisms or their metabolites is often linked to the storage or selling conditions, not to the fermentation/production process. For all the above mentioned reasons, this widespread Mediterranean fermented vegetable can be considered quite a safe product, if good hygiene and manufacturing practices are followed and appropriated levels of salt (>5%) and pH (<4.3) are obtained in the final products.

## Conflict of Interest Statement

The authors declare that the research was conducted in the absence of any commercial or financial relationships that could be construed as a potential conflict of interest.
